# Supplementary material for: CoxKAN: Kolmogorov-Arnold networks for interpretable, high-performance survival analysis
Source: Bioinformatics. 2025 Jul 21;41(8):btaf413. doi: 10.1093/bioinformatics/btaf413 (PMC12341683; doi:10.1093/bioinformatics/btaf413)
Supplement: btaf413_Supplementary_Data [file btaf413_supplementary_data.pdf]

# Appendix

## Hyperparameters

Supplementary Table 2 and 3-5 show the hyperparameters of DeepSurv and CoxKAN found for each experiment.

- Scale weights are initialized as $w_s = 1$ and $w_b = \frac{1}{n_{in}} + \text{Uniform}([-\xi_b, \xi_b])$, where $\xi_b$ is the "spline noise".
- Spline coefficients initialized as $c_i \sim \mathcal{N}(0, (\frac{\xi_s}{G})^2)$, where $\xi_s$ is "base noise".

The default auto-symbolic fitting for CoxKAN activation functions utilizes a library of 22 symbolic operators, which include: $\sin(x)$, $\tan(x)$, $\arctan(x)$, $\cosh(x)$, $e^x$, $e^{-x^2}$ $\log(x)$, $\tanh(x)$, $\arctan(x)$, sigmoid$(x)$, sgn$(x)$, $|x|$, $\sqrt{x}$, $\frac{1}{\sqrt{x}}$, $x$, $x^2$, $x^3$, $x^4$, $\frac{1}{x}$, $\frac{1}{x^2}$, $\frac{1}{x^4}$.

## Synthetic Data Generation

The simulated datasets were generated with 8000 training observations and 2000 testing observations. The death times were generated according to the exponential distribution: $T \sim \text{Exponential}(h(t, \mathbf{x}))$, where $h(t, \mathbf{x}) = 0.01e^{\theta(\mathbf{x})}$ is the hazard and $\theta(\mathbf{x})$ is custom log-partial hazard expression. We then generated censoring times $T_c$ uniformly in the range from 0 to the largest observed death time. The final observed times were then given by $Z = \min(T, T_c)$.

## Formulae Transformation

*Mixed function:*

$$\hat{\theta}_{KAN} = \tanh(5.1x_1) - \sin(6.3x_2 - 9.4) + x_3^2$$

where $-\sin(6.3x_2 - 9.4) \approx -\sin(2\pi x_2 - 3\pi) = \sin(2\pi x_2)$, showing consistency with the expected pattern.

*Euclidean function:*

$$\hat{\theta}_{KAN} = 4\sqrt{\begin{array}{l} x_3^2 + 0.8x_4^2 + 0.9\,(0.1 - x_1)^2 \\ + (0.1 - x_2)^2 - 0.5\,(x_1 + x_2 - 0.1)^2 \\ -0.7\,(x_3 + 0.7x_4 + 0.1)^2 + 0.6 \end{array}}$$

By multiplying this out and approximating the affine parameters, we recover the original formula:

$$\begin{aligned}\hat{\theta}_{KAN} &\approx 4\sqrt{\frac{1}{2}(x_1^2 - 2x_1x_2 + x_2^2 + x_3^2 - 2x_3x_4 + x_4^2)} \\ &= 2\sqrt{(x_1 - x_2)^2 + (x_3 - x_4)^2}.\end{aligned}$$

## CoxKAN Formulae (Clinical)

The learned formula for the SUPPORT dataset is given by:

$$\begin{aligned}\hat{\theta}_{KAN} = &\phi_{interact} - 0.0002 \cdot \text{age} + 0.003 \cdot \text{creatinine} + 0.04 \cdot \text{comorbidities} \\ &+ 0.9e^{-0.06(1-0.1\cdot\text{meanbp})^2} + 0.1\tanh(0.02 \cdot \text{hr} - 3) - 0.06\sin(0.08 \cdot \text{rr} + 0.2) \\ &+ 0.6e^{-572(1-0.02\cdot\text{temp})^2} + 0.0008 \cdot \text{sodium} + 0.03\tan(0.02 \cdot \text{wbc} - 4) \\ &+ \left\{\begin{array}{ll} 0.007 & \text{if male} \\ -0.01 & \text{if female} \end{array}\right\} + \left\{\begin{array}{ll} -0.03 & \text{if diabetes} \\ 0.0006 & \text{otherwise} \end{array}\right\} + \left\{\begin{array}{ll} 0.03 & \text{if dementia} \\ -0.0008 & \text{otherwise} \end{array}\right\} \\ &+ \left\{\begin{array}{ll} 0.003 & \text{if metastasis} \\ -0.01 & \text{if no cancer} \\ -0.0098 & \text{if cancer} \end{array}\right\},\end{aligned} \tag{1}$$

where the interaction term $\phi_{1,1,2}$ is expressed as:

$$\phi_{1,1,2}(x) = x - \sin(x + \tanh(\sin(x + 0.2)) - 0.8). \tag{2}$$

The learned formula for the GBSG dataset is given by:

$$\begin{aligned}\hat{\theta}_{KAN} = &+ \left\{\begin{array}{ll} -0.21 & \text{if hormonal therapy} \\ 0.28 & \text{otherwise} \end{array}\right\} + \left\{\begin{array}{ll} -0.07 & \text{if tumor size} \leq 20\,\text{mm} \\ 0.21 & \text{if } 20 < \text{tumor size} < 50\,\text{mm} \\ 0.48 & \text{if tumor size} \geq 50\,\text{mm} \end{array}\right\} \\ &+ \left\{\begin{array}{ll} -0.12 & \text{if pre-menopausal} \\ 0.23 & \text{if post-menopausal} \end{array}\right\} + 1.8\,(1 - 0.02 \cdot \text{age})^2 - 1.2e^{-0.02(\text{nodes}+0.4)^2} \\ &+ 0.1\cosh(0.002 \cdot \text{PGR} - 1.6) - 0.0007 \cdot \text{ER}.\end{aligned} \tag{3}$$

The learned formula for the METABRIC dataset is given by:

$$\begin{aligned}\hat{\theta}_{KAN} =& -0.24 \cdot \mathit{PGR} + 0.2\tanh(1.9 \cdot \mathit{MKI67} - 10) \\ &+ 0.7e^{-26(1-0.06\cdot \mathit{ERBB2})^2} - 1.7\sin(0.04 \cdot \text{age} - 9.5) \\ &+ \left\{ \begin{array}{ll} 0.1 & \text{if hormonal therapy} \\ 0.03 & \text{otherwise} \end{array} \right\} + \left\{ \begin{array}{ll} 0.01 & \text{if radiotherapy} \\ 0.18 & \text{otherwise} \end{array} \right\} \\ &+ \left\{ \begin{array}{ll} 0.6 & \text{if chemotherapy} \\ -0.05 & \text{otherwise} \end{array} \right\} + \left\{ \begin{array}{ll} 0.07 & \text{if ER positive} \\ -0.04 & \text{otherwise} \end{array} \right\}\end{aligned} \tag{4}$$

The learned formula for the FLCHAIN dataset is given by:

$$\begin{aligned}\hat{\theta}_{KAN} =& 0.09 \cdot \text{age} + \left\{ \begin{array}{ll} -0.047 & \text{if female} \\ 0.118 & \text{if male} \end{array} \right\} + 0.4\arctan(0.4 \cdot \text{year} - 737) + 0.04 \cdot \text{FLC}_{kappa} \\ &+ 0.3 \cdot \text{FLC}_{lambda} + 0.009 \cdot \text{FLC}_{\text{group}} + 2\arctan(0.5 \cdot \text{creatinine} - 0.9),\end{aligned} \tag{5}$$

The learned formula for the NWTCO dataset is given by:

$$\begin{aligned}\hat{\theta}_{KAN} =& \phi_{1,1,3} + \phi_{1,1,4} + 0.02 \cdot \text{age} + \left\{ \begin{array}{ll} -0.047 & \text{if FH (local)} \\ -0.014 & \text{if UH (local)} \end{array} \right\} \\ &+ \left\{ \begin{array}{ll} -0.22 & \text{if FH (central)} \\ 0.62 & \text{if UH (central)} \end{array} \right\} + \left\{ \begin{array}{ll} -0.47 & \text{if stage } = 1 \\ 0.04 & \text{if stage } = 2 \\ 0.35 & \text{if stage } = 3 \\ 0.78 & \text{if stage } = 4 \end{array} \right\} \\ &+ \left\{ \begin{array}{ll} 0.02 & \text{if } 3^{rd} \text{ study} \\ 0.01 & \text{if } 4^{th} \text{ study} \end{array} \right\} + \left\{ \begin{array}{ll} 0.2 & \text{if in subcohort} \\ -0.07 & \text{otherwise} \end{array} \right\}\end{aligned} \tag{6}$$

where the interaction term $\phi_{1,1,3}$ is expressed as:

$$\begin{aligned}\phi_{1,1,3} = -2.5\arctan\Bigg(2\Bigg[& + 0.03 \cdot \text{age} + \left\{ \begin{array}{ll} -0.1 & \text{if FH (local)} \\ -0.4 & \text{if UH (local)} \end{array} \right\} \\ &+ \left\{ \begin{array}{ll} 0.3 & \text{if FH (central)} \\ -0.4 & \text{if UH (central)} \end{array} \right\} + \left\{ \begin{array}{ll} 0.1 & \text{if stage } = 1 \\ -0.07 & \text{if stage } = 2 \\ -0.03 & \text{if stage } = 3 \\ -0.17 & \text{if stage } = 4 \end{array} \right\} \\ &+ \left\{ \begin{array}{ll} -0.2 & \text{if } 3^{rd} \text{ study} \\ 0.09 & \text{if } 4^{th} \text{ study} \end{array} \right\} + \left\{ \begin{array}{ll} 1 & \text{if in subcohort} \\ -0.3 & \text{otherwise} \end{array} \right\}\Bigg]\Bigg)\end{aligned} \tag{7}$$

and the interaction term $\phi_{1,1,4}$ is given by:

$$\begin{aligned}\phi_{1,4,1} = -\tanh\Bigg(0.7\Bigg[& + 0.006 \cdot \text{age} \\ &+ \left\{ \begin{array}{ll} 0.15 & \text{if favourable histology (instit)} \\ -0.7 & \text{if unfavourable histology (instit)} \end{array} \right\} \\ &+ \left\{ \begin{array}{ll} 0.1 & \text{if favourable histology (histol)} \\ 0.01 & \text{if unfavourable histology (histol)} \end{array} \right\} \\ &+ \left\{ \begin{array}{ll} -0.7 & \text{if stage } = 1 \\ -0.2 & \text{if stage } = 2 \\ 0.7 & \text{if stage } = 3 \\ 1.45 & \text{if stage } = 4 \end{array} \right\} \\ &+ \left\{ \begin{array}{ll} -0.6 & \text{if } 3^{rd} \text{ clinical study} \\ 0.5 & \text{if } 4^{th} \text{ clinical study} \end{array} \right\} \\ &+ \left\{ \begin{array}{ll} 1 & \text{if in subcohort} \\ -0.2 & \text{otherwise} \end{array} \right\}\Bigg]\Bigg)\end{aligned} \tag{8}$$

## CoxKAN Formula (Genomics)

CoxKAN derived the log-partial hazard formula for the GBM/LGG patient cohort as follows:

$$\begin{aligned}
\hat{\theta}_{KAN} = & -0.2 \cdot (\text{1p19q arm codeletion}) && (\sigma = 0.19) \\
& + e^{-0.2(-0.6\cdot(10\text{q}_{CNV})-1)^2} && (\sigma = 0.19) \\
& - 0.2 \cdot \mathit{IDH1}_{mut} && (\sigma = 0.17) \\
& - 0.06 \tan(0.4 \cdot \mathit{CARD11}_{CNV} + 8) && (\sigma = 0.16) \\
& - 0.08(0.6 \cdot \mathit{PTEN}_{CNV} + 1)^4 && (\sigma = 0.14) \\
& - 0.3 \sin(3 \cdot \mathit{JAK2}_{CNV} - 5) && (\sigma = 0.12) \\
& - 0.1 \cdot \mathit{CDKN2A}_{CNV} && (\sigma = 0.12) \\
& - 0.1 \sin(9 \cdot \mathit{CDKN2B}_{CNV} - 4) && (\sigma = 0.10) \\
& - 0.3 \sin(9 \cdot \mathit{EGFR}_{CNV} + 0.8) && (\sigma = 0.10) \\
& + \text{less significant terms},
\end{aligned} \tag{9}$$

CoxKAN derived the log-partial hazard formula for the BRCA patient cohort as follows:

$$\begin{aligned}
\hat{\theta}_{KAN} = & + 0.2 \cdot \mathit{KMT2C}_{mut} && (\sigma = 0.24) \\
& + 0.6 \sin(0.5 \cdot \mathit{HSPA8}_{RNA} - 7) && (\sigma = 0.18) \\
& - 2e^{-0.04(0.9\cdot \mathit{PLXNB2}_{RNA}+1)^2} && (\sigma = 0.17) \\
& - 2e^{-0.05(0.9\cdot \mathit{PGK1}_{RNA}+1)^2} && (\sigma = 0.15) \\
& - 0.14 \cdot \mathit{RYR2}_{mut} && (\sigma = 0.14) \\
& + 0.1 \cdot \mathit{DMD}_{mut} && (\sigma = 0.10) \\
& + 0.01 \cdot \mathit{TTN}_{mut} && (\sigma = 0.07) \\
& + \frac{0.4}{(1 - 0.1 \cdot \text{group_46}_{CNV})^2} && (\sigma = 0.06) \\
& + 0.9e^{-0.06(\mathit{H2BC5}_{RNA}-0.5)^2} && (\sigma = 0.05) \\
& - 0.3 \sin(0.5 \cdot \mathit{RPL14}_{RNA} + 5) && (\sigma = 0.05) \\
& + \text{less significant terms},
\end{aligned} \tag{10}$$

where group_46 is the median CNV of five highly correlated genes (*MRPS21P8, MRPS21P7, ZNF423, AC027348.2, AC027348.1*).
For the STAD dataset, CoxKAN predicted the following log-partial hazard:

$$\begin{aligned}
\hat{\theta}_{KAN} = & + 0.2 \tanh(\text{CALM2}_{RNA} - 0.4) && (\sigma = 0.15) \\
& - 0.1 \cdot \text{PRR15L}_{RNA} && (\sigma = 0.10) \\
& + 0.2 \cdot \text{TOMM20}_{RNA} && (\sigma = 0.09) \\
& - 0.09 \cdot \text{MUC16}_{mut} && (\sigma = 0.09) \\
& + 0.8 \arctan(0.4 \cdot \text{C3}_{RNA} + 0.2) && (\sigma = 0.08) \\
& - 0.1 \cdot \text{HNRNPK}_{RNA} && (\sigma = 0.08) \\
& - 0.2 \cdot \text{MISP}_{RNA} && (\sigma = 0.08) \\
& + \text{less significant terms}
\end{aligned} \tag{11}$$

For the KIRC dataset, CoxKAN predicted the following log-partial hazard:

$$\begin{aligned}
\hat{\theta}_{KAN} = & + 0.43 \cdot \text{MT1X}_{RNA} && (\sigma = 0.42) \\
& + 0.34 \cdot \text{DDX43}_{RNA} && (\sigma = 0.34) \\
& + 0.23 \cdot \text{CWH43}_{RNA} && (\sigma = 0.31) \\
& + 0.22 \cdot \text{CILP}_{RNA} && (\sigma = 0.31) \\
& - 0.24 \cdot \text{LOC153328}_{RNA} && (\sigma = 0.29) \\
& - 0.21 \cdot \text{CYP3A7}_{RNA} && (\sigma = 0.28) \\
& + \text{less significant terms},
\end{aligned} \tag{12}$$

For the LUAD dataset, CoxKAN predicted the following log-partial hazard:

$$
\begin{aligned}
\hat{\theta}_{KAN} = &- 1.056 \cdot \text{CLN6}_{RNA} && (\sigma = 0.53) \\
&- 1.0914 \cdot \text{DHRS1}_{RNA} && (\sigma = 0.75) \\
&+ 0.8042 \cdot \text{TFPI2}_{RNA} && (\sigma = 2.3) \\
&+ 0.7594 \cdot \text{DCP2}_{RNA} && (\sigma = 0.37) \\
&+ 0.7409 \cdot \text{FOXC1}_{RNA} && (\sigma = 1.0) \\
&+ \text{less significant terms,}
\end{aligned}
\tag{13}
$$

**Table 1.** Log-partial hazard functions and CoxKAN learned formulae for four synthetic datasets

| Dataset | Log-partial hazard | CoxKAN formula | Recovered |
|---|---|---|---|
| Gaussian | $5\exp(-2(x_1^2+x_2^2))$ | $4.98e^{-1.99(x_1^2+x_2^2)}$ | ✓ |
| Mixed | $\tanh(5x_1)+\sin(2\pi x_2)+x_3^2$ | $\tanh(5.1x_1)-\sin(6.3x_2-9.4)+x_3^2$ | ✓ |
| Euclidean | $2\sqrt{(x_1-x_2)^2+(x_3-x_4)^2}$ | $4\sqrt{x_3^2+0.8x_4^2+0.9(0.1-x_1)^2+(0.1-x_2)^2-0.5(x_1+x_2-0.1)^2-0.7(x_3+0.7x_4+0.1)^2+0.6}$ | ✓ |
| Complex | $\tanh(5(\log(x_1)+\lvert x_2\rvert))$ | $-\tanh\left(\frac{8.3}{(0.4x_1+1)^3}+3.0e^{-2.6x_2^2}-9.8\right)$ | × |

**Table 2.** Hyperparameters of DeepSurv.

| Hyperparameter | FLCHAIN | NWTCO | TCGA-STAD | TCGA-BRCA | TCGA-GBM/LGG | TCGA-KIRC | TCGA-LUAD |
|---|---|---|---|---|---|---|---|
| Shape | [8,5,1] | [6,9,1] | [148,19,19,1] | [168,15,1] | [320,19,1] | [362,18,18,1] | [429,13,1] |
| Early Stopping | True | False | False | True | False | True | False |
| Epochs | 300 | 135 | 131 | 300 | 114 | 300 | 12 |
| Learning Rate | 0.0067 | 0.008 | 0.002 | 0.001 | 0.001 | 0.006 | 0.003 |
| Batch Norm | True | True | True | True | True | False | True |
| Dropout | 0.12 | 0.15 | 0.27 | 0.15 | 0.11 | 0.14 | 0.34 |
| Weight Decay (L2) | 6.6e-8 | 4.7e-8 | 2.7e-7 | 4e-7 | 9e-5 | 3.2e-6 | 2.4e-8 |

**Table 3.** Hyperparameters of CoxKAN for Synthetic Datasets.

| Hyperparameter | Gaussian | Mixed | Euclidean | Complex |
|---|---|---|---|---|
| KAN Shape | [4,2,1] | [5,1] | [6,5,5,1] | [5,1] |
| Learning Rate | 0.035 | 0.01 | 0.01 | 0.1 |
| Early Stopping | False | False | True | False |
| Steps | 133 | 107 | (300) | 107 |
| Prune threshold | 0.03 | 0.03 | 0.045 | 0.03 |
| Grid Intervals | 4 | 5 | 4 | 5 |
| Base fn | linear | silu | linear | silu |
| Spline noise $\xi_s$ | 0.03 | 0.06 | 0.003 | 0.06 |
| Base noise $\xi_b$ | 0.13 | 0.14 | 0.16 | 0.14 |
| Reg $\lambda$ | 0.014 | 0.0001 | 0.01 | 0.0001 |
| Entropy Reg $\lambda_{ent}$ | 2 | 7 | 3 | 7 |
| Coefficient Reg $\lambda_{coef}$ | 0 | 0 | 2 | 0 |

**Table 4.** Hyperparameters of CoxKAN for Clinical Datasets.

| Hyperparameter | SUPPORT | GBSG | METABRIC | FLCHAIN | NWTCO |
|---|---|---|---|---|---|
| KAN Shape | [14,3,1] | [7,2,1] | [9,1] | [8,3,1] | [6,5,1] |
| Learning Rate | 0.015 | 0.0076 | 0.09 | 0.08 | 0.002 |
| Early Stopping | True | True | True | True | False |
| Steps | (300) | (300) | (300) | (300) | 147 |
| Prune threshold | 0.00007 | 0.045 | 0.035 | 0.001 | 0.02 |
| Grid Intervals | 3 | 3 | 3 | 3 | 5 |
| Base fn | linear | silu | silu | linear | linear |
| Spline noise $\xi_s$ | 0.11 | 0.09 | 0.1 | 0.12 | 0.15 |
| Base noise $\xi_b$ | 0.05 | 0.18 | 0.03 | 0.04 | 0.16 |
| Reg $\lambda$ | 0.005 | 0.0007 | 0.003 | 0.006 | 0.002 |
| Entropy Reg $\lambda_{ent}$ | 2 | 3 | 0 | 2 | 2 |
| Coefficient Reg $\lambda_{coef}$ | 4 | 2 | 4 | 1 | 2 |

**Table 5.** Hyperparameters of CoxKAN for Genomic Datasets.

| Hyperparameter | TCGA-STAD | TCGA-BRCA | TCGA-GBM/LGG | TCGA-KIRC | TCGA-LUAD |
|---|---|---|---|---|---|
| KAN Shape | [148,1] | [168,1] | [320,1] | [362,4,4,1] | [429,1] |
| Learning Rate | 0.005 | 0.03 | 0.014 | 0.014 | 0.0005 |
| Early Stopping | True | True | True | True | True |
| Steps | (300) | (300) | (300) | (300) | 500 |
| Prune threshold | 0.008 | 0.007 | 0.034 | 0.012 | 0.022 |
| Grid Intervals | 3 | 3 | 5 | 3 | 3 |
| Base fn | linear | silu | silu | linear | linear |
| Spline noise $\xi_s$ | 0.1 | 0.02 | 0.05 | 0.14 | 0.09 |
| Base noise $\xi_b$ | 0.01 | 0.009 | 0.04 | 0.11 | 0.18 |
| Reg $\lambda$ | 0.0004 | 0.013 | 0.01 | 0.01 | 0.01 |
| Entropy Reg $\lambda_{ent}$ | 10 | 14 | 0 | 3 | 1 |
| Coefficient Reg $\lambda_{coef}$ | 0 | 3 | 4 | 5 | 0 |

**Table 6.** Hyperparameters of Cox-nnet for Clinical Datasets.

| Hyperparameter | SUPPORT | GBSG | METABRIC | FLCHAIN | NWTCO |
|---|---|---|---|---|---|
| Learning Rate | 0.007 | 0.004 | 0.008 | 0.007 | 0.004 |
| Learning Rate Decay | 0.0002 | 5.62e-6 | 0.0002 | 0.0002 | 0.0001 |
| L2 Regularisation | 0.010 | 0.010 | 0.012 | 0.011 | 0.011 |
| Early Stopping | False | False | False | False | False |
| Steps | 306 | 441 | 188 | 306 | 396 |
| Dropout | 0.13 | 0.13 | 0.14 | 0.13 | 0.35 |

**Table 7.** Hyperparameters of Cox-nnet for Genomic Datasets.

| Hyperparameter | TCGA-STAD | TCGA-BRCA | TCGA-GBM/LGG | TCGA-KIRC | TCGA-LUAD |
|---|---|---|---|---|---|
| Learning Rate | 0.003 | 0.002 | 0.009 | 0.001 | 0.001 |
| Learning Rate Decay | 0.0002 | 5e-4 | 2.66e-6 | 8.79e-5 | 1.69e-6 |
| L2 Regularisation | 0.063 | 0.084 | 0.026 | 0.102 | 0.020 |
| Early Stopping | True | True | False | True | True |
| Steps | 500 | 500 | 243 | 500 | 500 |
| Dropout | 0.17 | 0.21 | 0.13 | 0.23 | 0.26 |

**Table 8.** Hyperparameters of SuMo for Clinical Datasets.

| Hyperparameter | SUPPORT | GBSG | METABRIC | FLCHAIN | NWTCO |
|---|---|---|---|---|---|
| Learning Rate | 0.005 | 0.002 | 0.002 | 0.006 | 0.002 |
| Hidden Layers | [100, 100, 12] | [67, 67, 67, 16] | [56, 56, 56, 49] | [44, 44, 32] | [81, 81, 48] |
| L2 Regularisation | 1.08e-6 | 1.65e-4 | 2.81e-8 | 2.08e-8 | 6.47e-7 |
| Early Stopping | True | False | True | True | False |
| Steps | 226 | 687 | 559 | 750 | 782 |
| Dropout | 0.16 | 0.12 | 0.11 | 0.13 | 0.10 |

**Table 9.** Hyperparameters of SuMo for Genomic Datasets.

| Hyperparameter | TCGA-STAD | TCGA-BRCA | TCGA-GBM/LGG | TCGA-KIRC | TCGA-LUAD |
|---|---|---|---|---|---|
| Learning Rate | 0.002 | 0.005 | 0.005 | 0.004 | 0.002 |
| Hidden Layers | [66, 66, 75] | [74, 76] | [20, 20, 20, 13] | [12, 12, 12, 12, 42] | [86, 86, 70] |
| L2 Regularisation | 5.07e-5 | 2.16e-9 | 7.08e-7 | 4.95e-7 | 2.44e-8 |
| Early Stopping | False | True | True | True | True |
| Steps | 968 | 425 | 492 | 111 | 763 |
| Dropout | 0.12 | 0.34 | 0.29 | 0.48 | 0.23 |
